# Mycobacterium abscessus Otitis Externa and Otitis Media Successfully Treated With Surgical Drainage: A Case Report

**DOI:** 10.7759/cureus.116824

**Published:** 2026-09-24

**Authors:** Taiki Sugaya, Takuya Kakuki, Ken-ichi Takano

**Affiliations:** 1 Otolaryngology - Head and Neck Surgery, Obihiro Kosei Hospital, Obihiro, JPN; 2 Otolaryngology - Head and Neck Surgery, Sapporo Medical University School of Medicine, Sapporo, JPN

**Keywords:** mycobacterium abscessus, nontuberculous mycobacteria, otitis externa, otitis media, surgical drainage

## Abstract

Nontuberculous mycobacteria (NTM) are environmental organisms that most commonly cause pulmonary disease; infections involving the external and middle ear are rare and can be diagnostically challenging. We report a case of chronic otitis externa and otitis media caused by *Mycobacterium abscessus* in a woman in her 30s. She presented to our department with an 18-month history of recurrent left aural fullness and otorrhea that had not responded to treatment at other facilities. At the referring hospital, she had marked swelling of the external auditory canal (EAC) with exposed bone, and nucleic acid amplification tests for *Mycobacterium tuberculosis*, *Mycobacterium avium*, and *Mycobacterium intracellulare* were negative. On presentation to our department, examination revealed marked edema and erythema of the EAC with purulent otorrhea. Despite repeated aural toilet and topical treatment, inflammation persisted. The tympanic membrane was thickened and perforated in the anteroinferior quadrant, and granulation-like tissue filled the middle ear. CT demonstrated soft-tissue opacification extending from the middle ear to the mastoid air cells without bony destruction. Because of persistent disease, mycobacterial culture of the otorrhea was performed at our department and identified *M. abscessus*. Oral levofloxacin and clarithromycin based on susceptibility testing provided limited improvement. The patient was therefore treated with IV imipenem and amikacin in addition to ongoing oral levofloxacin and clarithromycin, followed by mastoidectomy and tympanoplasty for drainage and source control. She remained free of recurrent otitis externa or otitis media, and CT one year after surgery showed complete resolution of the EAC thickening and middle-ear opacification. In refractory chronic otitis externa or media, particularly when routine testing is unrevealing, mycobacterial culture should be considered even when nucleic acid amplification tests performed at a referring institution are negative. Combined antimicrobial therapy and surgical drainage may achieve effective infection control in extensive or longstanding *M. abscessus* otologic infection.

## Introduction

Nontuberculous mycobacteria (NTM) are ubiquitous in natural environments, including soil and water systems, and are primarily recognized as causative pathogens of pulmonary infections [1]. Reports of NTM infections involving otorhinolaryngologic sites, such as the external and middle ear, are rare. Because these organisms are not detected by routine bacterial cultures, diagnosis can be challenging in patients with refractory otitis media. We report a case of otitis externa and otitis media caused by *Mycobacterium abscessus*.

## Case presentation

A 38-year-old woman presented with an 18-month history of recurrent aural fullness and otorrhea. She had initially visited a local clinic; however, her symptoms did not improve. Six months before presentation to our department, she was evaluated at another hospital, where marked swelling of the external auditory canal (EAC) and exposed bone were observed. Nucleic acid amplification tests, including PCR assays for *Mycobacterium tuberculosis* and NTM (*Mycobacterium avium *and *Mycobacterium intracellulare*), were negative. Tests for myeloperoxidase-antineutrophil cytoplasmic antibody and proteinase 3-antineutrophil cytoplasmic antibody were also negative. Further investigations did not reveal a definitive cause (Table 1). Despite regular outpatient aural toilet, her condition failed to improve, and she was referred to our department for further evaluation and treatment of chronic otitis externa.

**Table 1:** Results of laboratory and microbiological investigations during the initial evaluation. Leukopenia and mildly elevated C-reactive protein levels were noted; other hematological and biochemical parameters were within the reference ranges. Autoimmune serologies and nucleic acid amplification tests for the* M. tuberculosis* complex, and common NTM (*M. avium *and *M. intracellulare*) were negative. Routine bacterial culture yielded methicillin-susceptible *S. aureus*, whereas fungal culture was negative. NTM, nontuberculous mycobacteria

Hematological and biochemical findings	Result	Reference range	Interpretation
White blood cell count	3070/µL	3500-9700/µL	Low
Red blood cell count	4.37 × 10⁶/µL	3.76-5.16 × 10⁶/µL	Within reference range
Hemoglobin	12.6 g/dL	11.2-15.2 g/dL	Within reference range
Hematocrit	38.30%	34.3-45.2%	Within reference range
Platelet count	172 × 10³/µL	140-379 × 10³/µL	Within reference range
Mean corpuscular volume	88 fL	80-101 fL	Within reference range
Mean corpuscular hemoglobin	28.8 pg	26.4-34.3 pg	Within reference range
Mean corpuscular hemoglobin concentration	32.90%	31.3-36.1%	Within reference range
Basophils	0.70%	0.0-2.0%	Within reference range
Eosinophils	1.60%	0.0-7.0%	Within reference range
Lymphocytes	38.40%	18.0-50.0%	Within reference range
Monocytes	7.80%	1.0-8.0%	Within reference range
Neutrophils	51.50%	42.0-74.0%	Within reference range
Erythroblasts	0.0/100 white blood cells	0	Within reference range
Activated partial thromboplastin time	30.1 seconds	26.0-38.0 seconds	Within reference range
Prothrombin time	11.3 seconds	10.0-13.0 seconds	Within reference range
Prothrombin time activity	102.80%	80.0-120.0%	Within reference range
Prothrombin time-international normalized ratio	0.99	0.90-1.13	Within reference range
Aspartate aminotransferase	18 U/L	10-40 U/L	Within reference range
Alanine aminotransferase	14 U/L	5-45 U/L	Within reference range
Lactate dehydrogenase	353 U/L	220-430 U/L	Within reference range
Gamma-glutamyl transferase	12 U/L	≤48 U/L	Within reference range
Creatinine	0.80 mg/dL	0.46-0.82 mg/dL	Within reference range
Blood urea nitrogen	10.1 mg/dL	8.0-20.0 mg/dL	Within reference range
Hemoglobin A1c	5.20%	4.6-6.2%	Within reference range
C-reactive protein	0.37 mg/dL	≤0.30 mg/dL	High
Rapid plasma reagin	Negative	Negative	Negative
Treponema pallidum antibody	Negative	Negative	Negative
Antinuclear antibody	<1:40 titer	<1:40	Negative
Rheumatoid factor	5 IU/mL	≤15 IU/mL	Within reference range
Proteinase 3-antineutrophil cytoplasmic antibody	<0.5 IU/mL	<2.0 IU/mL	Negative
Myeloperoxidase-antineutrophil cytoplasmic antibody	<0.5 IU/mL	<3.5 IU/mL	Negative
Mycobacterial testing results	-	-	-
*Mycobacterium tuberculosis*complex nucleic acid amplification test	Negative	Negative	Negative
NTM identification by PCR	-	-	-
Mycobacterium avium	Negative	Negative	Negative
Mycobacterium intracellulare	Negative	Negative	Negative
Routine bacterial and fungal culture results	-	-	-
Methicillin-sensitive *Staphylococcus aureus* detected; no fungal growth detected	-	-	-

At presentation, erythema and swelling of the anterior and posterior walls of the EAC were noted. The canal was filled with otorrhea, precluding visualization of the tympanic membrane (Figure 1A). Microbiological culture yielded methicillin-susceptible *Staphylococcus aureus*; fungal cultures were negative. A biopsy specimen obtained from the EAC showed nonspecific inflammatory changes, with no evidence of vasculitis or malignancy.

**Figure 1:**
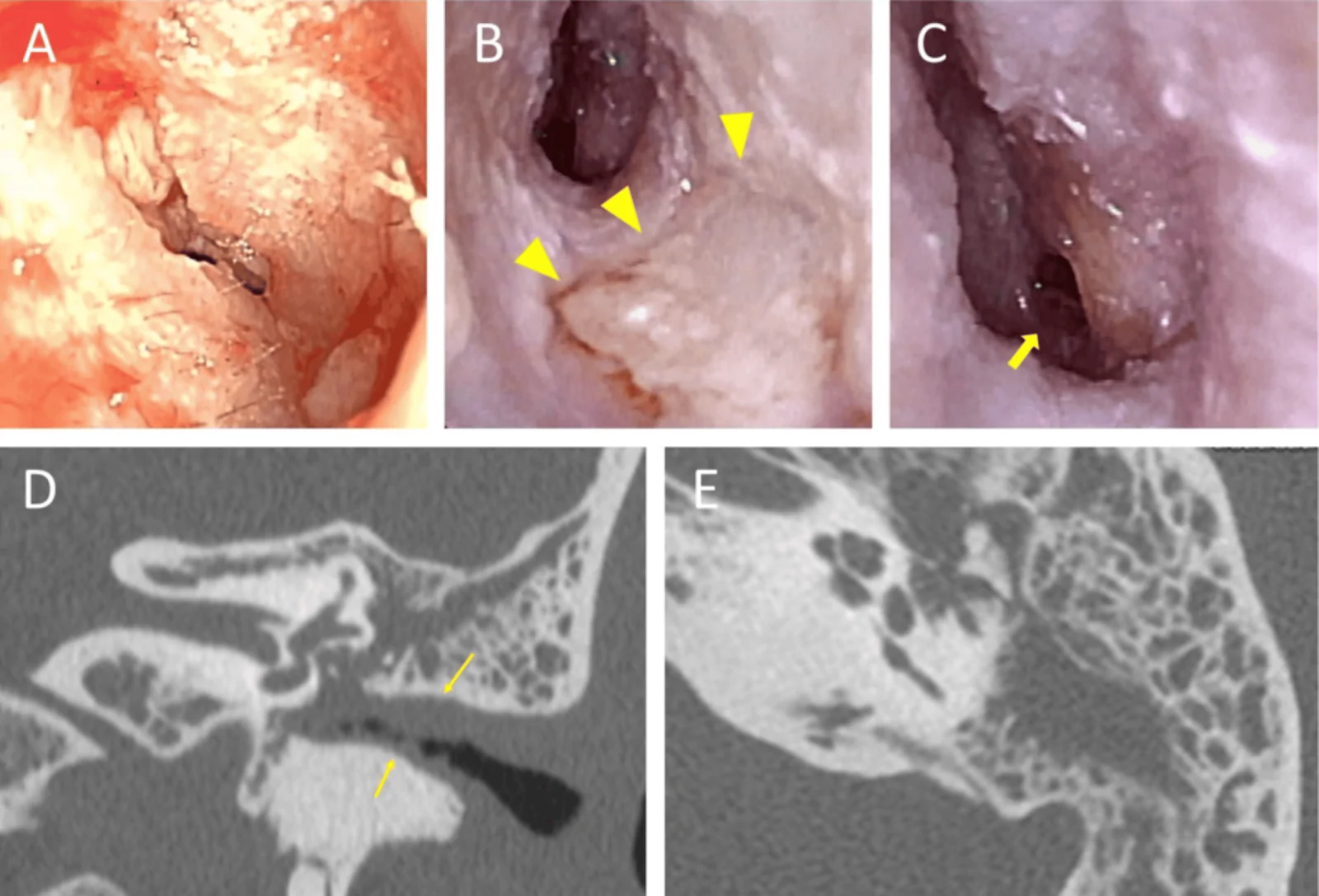
Otoscopic and CT findings before surgical treatment. (A) At the initial visit, marked erythema and swelling of the anterior and posterior walls of the left EAC were observed. The EAC was filled with otorrhea, and the tympanic membrane could not be visualized. (B) After two months of topical treatment and regular aural toilet, exposed bone was observed in the EAC (▶). (C) The tympanic membrane was thickened, with a perforation in the anteroinferior quadrant (→). (D, E) Coronal and axial temporal bone CT images obtained two months after the initial visit demonstrate thickening of the EAC (→) and soft-tissue opacification extending from the middle-ear cavity to the mastoid air cells. No destruction of the EAC, ossicles, or skull base was observed. EAC, external auditory canal

During the first two months after presentation, regular aural toilet and topical levofloxacin produced transient improvement in the otitis externa; however, inflammatory findings persisted. Exposed bone in the EAC, thickening of the tympanic membrane, and a perforation in the anteroinferior quadrant were subsequently observed (Figure 1B, 1C). Temporal bone CT, performed two months after presentation, showed thickening of the EAC and soft-tissue opacification extending from the middle-ear cavity to the mastoid air cells. No bone destruction was observed in the EAC, ossicles, or skull base (Figure 1D, 1E).

Because the otologic findings did not improve, an acid-fast bacilli culture of the otorrhea was obtained three months after presentation and yielded *M. abscessus*. Based on the antimicrobial susceptibility results, oral levofloxacin and clarithromycin were initiated. However, after two months of oral therapy, the clinical response remained limited. Therefore, five months after presentation to our department, the patient was admitted for surgical drainage. Beginning on the day before surgery, IV imipenem and amikacin were added to the ongoing oral levofloxacin and clarithromycin regimen. Mastoidectomy and tympanoplasty were then performed to drain the infected focus.

Surgery was performed under general anesthesia. A postauricular incision was made on the left side, and the surface of the temporal bone was exposed. The tympanic cavity was first inspected through the EAC. The EAC skin was elevated, and areas of skin with granulomatous degeneration were resected. After the tympanic membrane was elevated, the malleus and incus were identified, and the tympanic cavity was found to be filled with granulation tissue (Figure 2A). Mastoidectomy was then initiated. Granulation-like tissue was identified and removed from the mastoid cavity. The incus was identified, and granulation tissue filling the epitympanum was removed (Figure 2B). A posterior tympanotomy was also performed, and granulation tissue in the tympanic cavity was removed as completely as possible. After irrigation with saline (Figure 2C), an inferiorly based flap was positioned over the exposed EAC bone. A drain was placed in the mastoid cavity, and the wound was closed.

**Figure 2:**
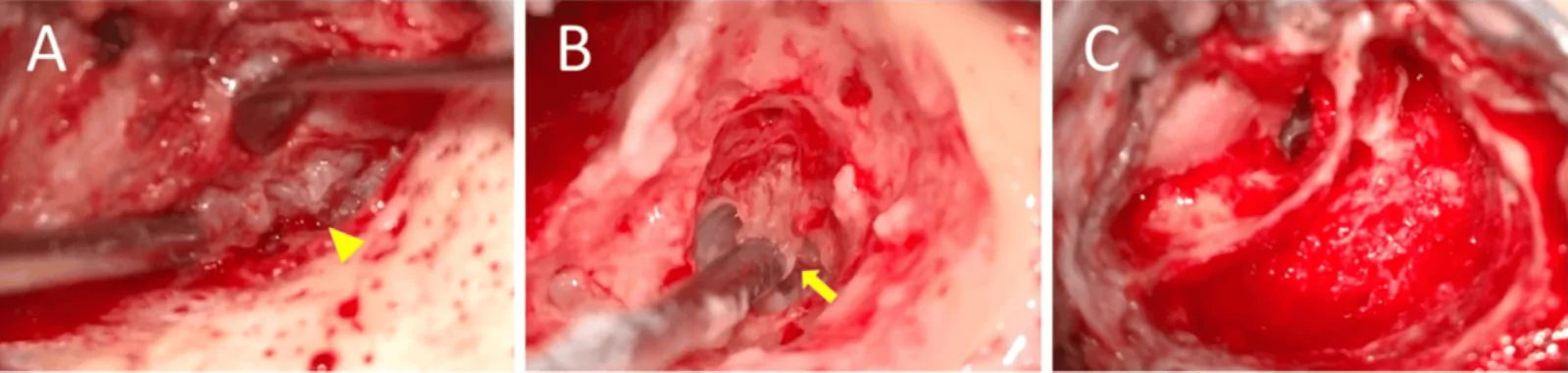
Intraoperative findings during mastoidectomy and tympanoplasty. (A) After elevation of the tympanic membrane, the tympanic cavity was found to be filled with granulation tissue (▶). (B) Granulation-like tissue filling the epitympanum (→) and mastoid cavity was removed during mastoidectomy. (C) The tympanic cavity and mastoid cavity after removal of the granulation tissue and irrigation with saline.

The postoperative course was uneventful. The drain was removed on postoperative day 3, and the patient was discharged on postoperative day 4. IV imipenem was continued until discharge, for a total of six days. IV amikacin, to which the isolate was susceptible, was continued for three additional days after discharge and was discontinued after a total of 10 days of treatment. Oral levofloxacin and clarithromycin were continued after discharge. Although a tympanic membrane perforation persisted, inflammation of the EAC resolved, and no recurrence was observed (Figure 3A). CT performed one year after surgery confirmed resolution of EAC thickening and middle-ear opacification (Figure 3B, 3C). Oral levofloxacin and clarithromycin were therefore discontinued after one year of postoperative therapy, corresponding to a total oral antimicrobial treatment duration of 14 months. At two years after surgery, no recurrence was observed. During an additional 28 months of follow-up, no recurrence was reported.

**Figure 3:**
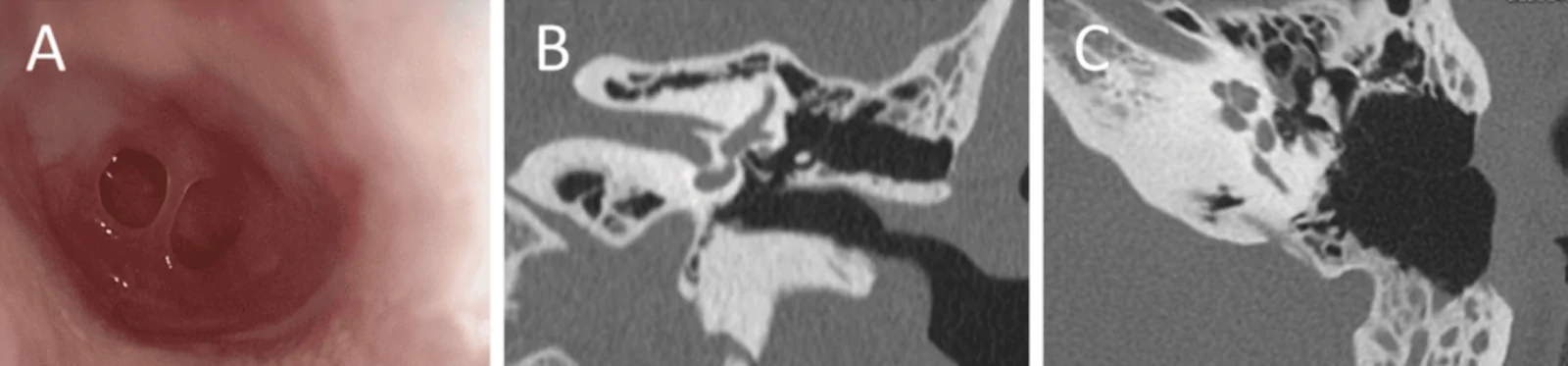
Postoperative findings. (A) Otoscopic examination after surgery shows resolution of inflammation in the left EAC, although a tympanic membrane perforation persisted. (B, C) Axial and coronal temporal bone CT images obtained one year after surgery demonstrate resolution of EAC thickening and soft-tissue opacification of the middle ear and mastoid air cells. EAC, external auditory canal

## Discussion

NTM comprise acid-fast mycobacteria other than* M. tuberculosis* complex and *Mycobacterium leprae*, with more than 172 species currently recognized. Although pulmonary disease is the most common manifestation of NTM infection, lymph node disease, skin and soft-tissue infection, bone and joint infection, and disseminated disease can also occur [1]. NTM are widely distributed in natural environments, including water systems, soil, and animals, and species distribution and disease incidence vary by region and country. For example,* M. avium* complex (MAC) is the most frequently isolated NTM in Western countries, European countries, and East Asian countries. In contrast, *M. abscessus*, *Mycobacterium fortuitum*, and* M. intracellulare *have been reported as the predominant NTM species in Saudi Arabia, whereas* M. fortuitum* predominates in India [1]. Although the patient owned a dog as a pet, she had no known environmental exposure to water systems or soil. Therefore, the route of infection could not be determined.

We searched PubMed for English-language, freely accessible reports published from January 2011 through August 2026 using combinations of the terms “nontuberculous mycobacteria” and “otitis.” Reports providing sufficient individual patient-level data were included, and duplicate reports were excluded. Among the included reports, 105 patients with 111 NTM isolates were identified.* M. abscessus* accounted for 87 isolates (78.4%), making it the most frequently reported causative organism in this dataset (Table 2) [2-16]. The reason why *M. abscessus* is common in NTM otitis media remains unknown. However, *M. abscessus* is an environmental organism known to cause pulmonary infection, skin and soft-tissue infection, and healthcare-associated device infection [17]. Therefore, disruption of the skin barrier of the auricle or EAC, water exposure of the EAC, or tympanostomy tube placement may facilitate bacterial entry through the EAC. In pulmonary NTM disease, MAC is the most common causative group, in contrast to* M. abscessus*, which is the principal pathogen in NTM otologic infection. This difference in species distribution suggests that NTM otologic infection may result from infection through the EAC rather than spread from pulmonary NTM disease through the airway and Eustachian tube.

**Table 2:** Summary of cases of NTM otitis media identified in freely accessible English-language reports retrieved from PubMed (January 2011 through August 2026), including the present case. Reports providing sufficient individual patient-level data were included, and duplicate reports were excluded. A total of 105 patients with 111 reported NTM isolates were identified. *M. abscessus* accounted for 87 of 111 isolates (78.4%), and surgical drainage was performed in 85 of 105 patients (81.0%). Because more than one NTM species was reported in some patients, isolate-based species counts do not sum to the total number of patients. MAC, *Mycobacterium avium *complex; NTM, nontuberculous mycobacteria

Reference no.	Year	No. of cases	Age, years (range)	Sex, M:F	Mycobacterial species, n	*Mycobacterium abscessus*	*Mycobacterium fortuitum*	MAC	*Mycobacterium massiliense*	*Mycobacterium intracellulare*	*Mycobacterium chelonae*	Others	Surgical drainage, n
[2]	2011	1	54	0:01	-	-	-	-	-	1	1	-	1
[3]	2012	2	53-58	2:00	-	-	-	2	-	-	-	-	1
[4]	2013	1	22	0:01	-	-	-	-	-	1	-	1	1
[5]	2014	1	64	0:01	-	1	-	-	-	-	-	-	1
[6]	2015	1	2	1:00	-	1	-	-	-	-	-	-	1
[7]	2015	16	1-52	11:05	-	12	2	2	-	-	-	-	13
[8]	2016	22	42-81	10:12	-	18	2	-	-	1	-	1	20
[9]	2018	1	10	1:00	-	1	-	-	1	-	-	-	1
[10]	2020	4	7-15	2:02	-	4	-	-	-	-	-	-	4
[11]	2020	8	1-6	6:02	-	7	-	1	-	-	-	-	3
[12]	2021	3	29-71	2:01	-	3	-	-	-	-	-	-	3
[13]	2022	10	5-15	7:03	-	10	-	-	-	-	-	-	9
[14]	2023	3	40-59	0:03	-	2	-	-	-	-	-	1	3
[15]	2024	1	27	0:01	-	1	-	-	-	-	-	-	0
[16]	2025	30	7-80	14:16	-	26	3	2	-	-	1	2	23
Our case	2026	1	38	0:01	-	1	-	-	-	-	-	-	1
Total	-	105	-	-	-	87	7	5	3	2	2	5	85

Most reported NTM otologic infections involve otitis media. Our patient was referred with refractory chronic otitis externa and had marked EAC swelling and exposed bone at the initial visit; thus, otitis externa was initially considered the predominant lesion. In patients with severe otitis externa, the differential diagnosis includes EAC carcinoma, necrotizing otitis externa, and malignant lymphoma. NTM infection may therefore be overlooked. The range of organisms detected by nucleic acid amplification testing for NTM varies according to the laboratory and assay used. Thus, while MAC may be rapidly identified, identification of *M. abscessus*, particularly at the subspecies level, may require additional molecular testing or testing at a reference laboratory. When NTM infection is suspected in refractory otitis externa or otitis media, acid-fast bacilli culture should be performed in addition to routine culture, and positive isolates should undergo species identification [18]. Because NTM are ubiquitous environmental organisms, contamination or colonization of clinical specimens must also be considered. A single positive culture should not be regarded as definitive evidence of causation; repeated isolation from independent specimens and consistency with the clinical findings and treatment course should be assessed [19].

*M. abscessus* is a rapidly growing NTM that is difficult to treat because of intrinsic and acquired resistance to many antimicrobial agents. In particular, inducible macrolide resistance caused by a functional erm(41) gene and constitutive macrolide resistance associated with mutations in the rrl gene affect treatment strategy and prognosis [20]. Antimicrobial therapy should be individualized based on the species and subspecies, drug susceptibility testing results, including macrolide susceptibility, site and severity of infection, and host factors. In general, multidrug treatment combines injectable agents such as amikacin, imipenem (or cefoxitin), or tigecycline with agents such as a macrolide, clofazimine, or linezolid [20].

The effectiveness of surgery for NTM otologic infection has not been established. Nevertheless, surgical drainage by mastoidectomy and/or tympanoplasty was performed in 85 of 105 reported cases (81.0%) identified in our review (Table 2). Although some cases have improved with medical treatment alone [15], the drug-resistance characteristics of *M. abscessus *suggest that surgical removal or drainage of infected tissue is frequently required. In the present case, otitis externa was present, and the disease duration approached two years from symptom onset. After the diagnosis was established, early mastoidectomy for drainage was performed in addition to antimicrobial therapy, resulting in prompt and sustained infection control. Although the indications and optimal timing for surgical drainage remain undefined, early surgical drainage may be considered in patients with marked inflammation accompanied by otitis externa or a prolonged disease course, as in this case.

## Conclusions

We report a case of otitis externa and otitis media caused by *M. abscessus*. Otologic NTM infection may result from transcanal infection through a tympanic membrane perforation. In patients with refractory otitis externa or otitis media, acid-fast bacilli culture should be performed in addition to routine bacterial culture, and NTM infection should be included in the differential diagnosis. In cases with severe otitis externa, such as the present case, EAC carcinoma and necrotizing otitis externa must also be considered, which may further complicate the diagnosis. In addition, *M. abscessus*, rather than MAC, frequently causes NTM otologic infection; therefore, clinicians should recognize that this pathogen may not be detected by PCR assays used at some institutions. NTM otologic infections, particularly those caused by *M. abscessus*, are often refractory to antimicrobial therapy. When conservative treatment produces an inadequate response, prompt surgical drainage with mastoidectomy and tympanoplasty should be considered.
